# Artificial Intelligence for Neonatal Sepsis Prediction: A Review of Advances, Clinical Integration, and Future Prospects

**DOI:** 10.7759/cureus.110241

**Published:** 2026-06-04

**Authors:** Joyce O Ndubuisi, Igboanude Chimaoge Henry, Oranu G Ibekie, Winifred Oniah, Gandu T Purity, Johnbosco C Nnamani, Oiku M Omofuma, Ubochioma D Chimdike, Nwachi Divine, Izuchukwu Jonathan Obidiegwu, Umeozulu Jessica Ifunanya, Prestige Okonkwo, Osinigwe Ibekie, Nwachukwu Chinenye Benita, Patrick Ashinze

**Affiliations:** 1 Medicine and Surgery, College of Medical Sciences, Chukwuemeka Odumegwu Ojukwu University, Uli, NGA; 2 Medicine, Enugu State University Teaching Hospital, Enugu, NGA; 3 Medicine, Indiana University School of Medicine Northwest, Gary, USA; 4 Medicine, Methodist Hospitals, Gary, USA; 5 Medicine, Tambov State University, Tambov, RUS; 6 Medicine, Enugu State University of Science and Technology (ESUST), Enugu, NGA; 7 Medicine, Alex Ekwueme Federal University Teaching Hospital, Abakaliki, NGA; 8 Medicine, Imo State University Teaching Hospital, Orlu, NGA; 9 Internal Medicine, Babcock University Teaching Hospital, Ilishan-Remo, NGA; 10 Obstetrics and Gynecology, University of Nigeria Teaching Hospital, Enugu, NGA; 11 Medicine, Chukwuemeka Odumegwu Ojukwu University Teaching Hospital, Awka, NGA; 12 Medicine, Chukwuemeka Odumegwu Ojukwu University, Awka, NGA; 13 Medicine, Northeast Ohio Medical University (NEOMED), Rootstown, USA; 14 Clinical Sciences, University of Ilorin, Ilorin, NGA

**Keywords:** artificial intelligence, clinical decision support, deep learning, early-onset sepsis, late-onset sepsis, machine learning, neonatal intensive care unit, neonatal sepsis, predictive modelling

## Abstract

Neonatal sepsis (NS) remains a cardinal cause of morbidity and mortality worldwide, disproportionately affecting neonates in low- and middle-income countries (LMICs). Blood culture, the current diagnostic reference standard, is burdened by prolonged turnaround times and the pervasive phenomenon of culture-negative sepsis, a limitation compounded by antenatal antibiotic exposure in mothers rather than representing an intrinsically poor tool. Conventional biomarkers, including C-reactive protein (CRP), procalcitonin, and lactate, are time-dependent in their rise and require integration with clinical context and professional experience to yield meaningful diagnostic value. These limitations create a persistent window of diagnostic uncertainty during which adverse outcomes may compound, and it is precisely in this gap that artificial intelligence (AI) offers supplementary clinical value.

This structured narrative review synthesizes published literature on AI, machine learning (ML), and deep learning (DL) applications for NS prediction. Evidence was drawn from peer-reviewed studies identified through a structured search of the PubMed, Google Scholar, and IEEE Xplore databases, retrieving approximately 430 records in total, of which 44 studies met inclusion criteria following title and abstract screening and full-text review. The review incorporated both early-onset sepsis (EOS) and late-onset sepsis (LOS) prediction models, with particular attention to algorithm design, validation methodology, and clinical integration.

AI-based models demonstrate substantial discriminative performance when evaluated alongside but not in place of existing clinical tools. Ensemble tree-based classifiers for EOS, including CatBoost (Categorical Boosting) and XGBoost (Extreme Gradient Boosting), report area under the receiver operating characteristic curves* *(AUROCs) exceeding 0.95 in retrospective cohorts (95% confidence intervals not reported in source studies). Continuous physiological monitoring models for LOS achieve AUROC values of 0.81 to 0.90, with early warning signals emerging 6 to 12 hours before clinical deterioration. The HeRO (Heart Rate Observation) monitor, a validated heart rate characteristics system, produced a statistically significant 22% relative reduction in mortality in a prospective randomized controlled trial. Nevertheless, most studies remain retrospective, single-center, and deficient in external validation, limiting confidence in real-world generalizability.

AI holds transformative potential as a clinical decision-support adjunct for NS prediction, offering early risk stratification that complements rather than supplants the clinician’s judgment and existing diagnostic tools. Realizing this potential demands prospective multicentric validation, context-sensitive model development for LMICs, advances in explainable AI, and robust regulatory and ethical frameworks. The goal is an equitable, globally applicable partnership between AI and the clinician.

## Introduction and background

Neonatal sepsis (NS) is defined as a dysregulated host response to a systemic viral, bacterial, or fungal infection occurring within the first 28 days of life--a definition that reflects the modern understanding of sepsis as an exaggerated immune reaction with cascading release of inflammatory mediators, rather than infection per se [1,2]. The condition is conventionally stratified by the timing of clinical onset: early-onset sepsis (EOS) manifests at or before 72 hours of age, whereas late-onset sepsis (LOS) is defined as sepsis presenting at or after 72 hours. A third classification, very late-onset NS (VLOS), describes infections typically emerging after 30 days of continuous hospitalization in the neonatal intensive care unit (NICU) [2,3].

Globally, NS remains among the most consequential drivers of neonatal mortality, accounting for approximately 8% of all neonatal deaths [4,5]. The burden is strikingly unequal: prevalence estimates approach 29.2-29.9% in LMICs in certain multi-country facility-based studies [6], compared with 9-15% in high-income countries. Incidence ranges from 1 to 4 per 1,000 live births in well-resourced settings, but surges to 49-170 per 1,000 in LMICs, where case fatality rates approach 24% [6-8]. These epidemiological gradients underscore the intersection of neonatal vulnerability with healthcare system capacity.

Despite significant advances in neonatal medicine, early recognition of NS remains clinically elusive. The signs of infection in neonates are inherently non-specific, frequently resembling other common conditions, such as respiratory distress syndrome or birth asphyxia, and overt clinical deterioration often lags behind the onset of pathophysiological derangement [9,10]. This diagnostic lag carries direct mortality implications: each hour of delayed treatment increases the probability of progression to septic shock and end-organ injury [10,11].

The diagnostic workup for NS, while well-established, carries important practical limitations. Blood culture--the reference standard--may take 24-72 hours to yield results, and culture negativity is frequently encountered when mothers have received intrapartum antibiotic prophylaxis, not because the tool is inherently poor, but because prior antibiotic exposure suppresses organism growth [11-14]. This diagnostic gap between clinical suspicion and laboratory confirmation compels clinicians toward empirical antibiotic prescribing, with attendant risks of antimicrobial resistance, microbiome disruption, and prolonged NICU admission [15-17]. Biochemical markers, such as CRP and procalcitonin, are valuable adjuncts but are time-dependent; procalcitonin, for instance, begins rising two to four hours after infection onset and only achieves diagnostic utility when interpreted by experienced clinicians integrating clinical risk factors [18-20].

It is against this backdrop that artificial intelligence (AI) offers supplementary clinical value. Rather than replacing existing diagnostic tools or the judgment of experienced clinicians, AI-based systems function best as an additional layer of pattern recognition, synthesizing continuous physiological data, biomarker trajectories, and perinatal risk factors into evolving risk estimates that support, rather than supplant, clinical decision-making [18]. This review examines the current state of AI-based NS prediction, evaluates pathways for safe clinical integration, identifies existing translational barriers, and charts future directions for equitable global implementation.

## Review

Methodology

This structured narrative review was conducted to synthesise published evidence on the application of AI, machine learning (ML), and deep learning (DL) to NS prediction. The review is explicitly framed as a narrative synthesis and was not registered prospectively, consistent with standard practice for narrative reviews. The word 'systematic' has been removed from the search description throughout to accurately reflect the methodology employed; this review does not claim PRISMA-compliant systematic review procedures, and no PRISMA flow diagram, dual independent screening, or formal risk-of-bias assessment was undertaken. Literature management and data synthesis were conducted using standard office software.

Search Strategy and Databases

A structured literature search was conducted across three electronic databases: PubMed (National Library of Medicine), Google Scholar (Google LLC), and IEEE Xplore (Institute of Electrical and Electronics Engineers). The search encompassed studies published between January 2016 and January 2026--a period selected to capture the post-MIMIC-III (Medical Information Mart for Intensive Care (third edition)) era. The following Boolean query was applied across all databases: ("artificial intelligence" OR "machine learning" OR "deep learning" OR "neural network") AND ("neonatal sepsis" OR "early-onset sepsis" OR "late-onset sepsis" OR "neonatal infection") AND ("prediction" OR "detection" OR "classification" OR "model"). The search retrieved approximately 430 records in total (PubMed: approximately 180 records; Google Scholar: approximately 170 records; IEEE Xplore: approximately 80 records). Following deduplication and application of inclusion and exclusion criteria through title and abstract screening and full-text review, 44 studies were included in the final narrative synthesis. Embase, Cochrane CENTRAL, and Scopus were not searched; this is acknowledged as a limitation consistent with the narrative framing of this review.

Inclusion and Exclusion Criteria

Studies were considered eligible for inclusion if they: (i) reported the development, validation, or clinical evaluation of an AI, ML, or DL model for NS prediction or detection; (ii) were published in English in peer-reviewed journals; (iii) included neonates (age 0-28 days) as the primary study population; and (iv) reported at least one quantitative performance metric (e.g., area under the receiver operating characteristic curve (AUROC), sensitivity, specificity, F1 score). Studies were excluded if they: (i) were case reports, conference abstracts without full-text data, letters to the editor, or editorial commentaries; (ii) focused exclusively on sepsis in older paediatric or adult populations without a neonatal-specific analysis; (iii) used AI exclusively for treatment optimization without a diagnostic or predictive component; or (iv) were duplicate publications of the same dataset without new methodological contribution.

Study Selection and Data Extraction

Following the application of inclusion and exclusion criteria, data were extracted narratively with attention to algorithm type, training dataset characteristics, input feature domains, validation methodology, primary performance metrics, and key limitations. Given the heterogeneity of outcome definitions and modelling approaches across included studies, formal meta-analytic pooling was not attempted.

Discussion

Diagnostic Dilemmas and the Imperative for Innovation

The diagnosis of NS is among the most challenging clinical tasks in neonatology, characterized by diagnostic uncertainty that stems from the convergence of non-specific symptomatology, imperfect reference standards, and the inherent physiological vulnerability of the neonatal host.

Limitations of clinical non-specificity: Neonates seldom mount conspicuous inflammatory responses. Early sepsis may manifest only as subtle changes: poor feeding, temperature instability, mild tachycardia, or apnea, all of which overlap substantially with conditions, including necrotizing enterocolitis, respiratory distress syndrome, and intracranial pathology [10]. The primary clinical challenge is not differential diagnosis per se, since diagnostic uncertainty almost invariably prompts antibiotic initiation; rather, it is the lack of structured, continuous clinical monitoring, particularly in the postpartum period, which allows early deterioration to go undetected. AI, as a continuous monitoring layer, is uniquely positioned to address this specific gap.

Limitations of blood culture: Blood culture remains the gold standard for confirming NS, offering the dual advantage of organism identification and antimicrobial susceptibility profiling [11]. Its practical limitations are well-documented and are best understood as operational rather than intrinsic. Sensitivity is constrained, with detection rates as low as 25-40%, a deficiency attributable in part to the small blood volumes obtainable from preterm neonates [12], and in significant part to the common scenario in which mothers have received intrapartum antibiotic prophylaxis, suppressing organism growth in the culture bottle [11]. Results require 24 to 72 hours, precluding timely targeted therapy. Contamination rates range from 2.6% to 18%, resulting in clinically misleading false-positive results that may trigger unnecessary antibiotic courses [13,14].

Culture-negative sepsis, in which clinical and hematological evidence strongly suggests infection despite sterile cultures, represents a particularly vexing diagnostic scenario. Clinicians confronted with such cases must choose between withholding antibiotics from a genuinely infected neonate and prolonged empirical exposure in an uninfected one. The latter carries established consequences: antimicrobial resistance selection, disruption of the gut microbiome, invasive fungal infection, and prolonged hospitalization [15-17]. The role of AI is not to replace blood culture as a diagnostic tool. Blood culture identifies organisms and directs therapy in a way no AI model currently can. The role is to provide earlier risk stratification that can guide the timing and urgency of workup initiation.

Biomarker limitations: CRP, the most widely measured biomarker, rises later in the inflammatory cascade, lacks infection specificity, and may be elevated in non-infectious conditions such as birth asphyxia and meconium aspiration [18,19]. Biochemical markers are valuable adjuncts to blood culture and clinical assessment. Their limitations are primarily temporal rather than diagnostic: they do not indicate absent sensitivity, but rather a physiological time-lag in their rise. Procalcitonin, for instance, begins to rise two to four hours after infection onset and achieves useful predictive value when interpreted by an experienced clinician integrating clinical presentation and perinatal risk factors [20]. Lactate reflects tissue hypoxia rather than infection per se, and its elevation may be explained by haemodynamic instability or metabolic disturbances unrelated to sepsis.

Interleukin-6 (IL-6) has demonstrated elevated concentrations in both EOS and LOS and, when combined with CRP and procalcitonin, improves diagnostic accuracy over any single marker [21,22]. No single biomarker combination achieves sufficient standalone accuracy to safely rule in or rule out NS; this is not a failure of the markers but a reflection of the complexity of the neonatal immune response. AI-based approaches are positioned to complement this biomarker panel by integrating physiological trajectory data with biochemical trends over time [23].

Artificial Intelligence and Machine Learning: Conceptual Foundations

AI is a broad term encompassing computational systems designed to perform tasks that conventionally require human cognitive capabilities, including pattern recognition, inference, and decision-making. Within this domain, ML constitutes a subset of AI in which algorithms learn predictive models directly from data rather than relying on explicitly programmed rules, iteratively improving performance by minimizing prediction error. DL extends this paradigm further by employing multilayered artificial neural network architectures capable of autonomously extracting hierarchical, abstract features from high-dimensional inputs [24].

ML approaches are broadly categorized by their learning paradigm. Supervised learning algorithms are trained on labeled datasets, examples in which the outcome of interest (sepsis or no sepsis) is known, to learn a classification or regression function applicable to new cases. Unsupervised learning, in contrast, operates without predefined outcome labels and aims to discover latent structure within the data, such as identifying clinically meaningful sub-phenotypes of NS patients with shared physiological signatures [25]. Such clustering may reveal, for instance, a subgroup characterized by early hematological abnormalities distinct from one defined predominantly by hemodynamic instability, with implications for personalized risk stratification.

A critical distinction exists between ML and classical statistical modeling. Traditional logistic regression is a parametric, hypothesis-driven approach in which the investigator specifies predictor variables and estimates odds ratios to support causal inference. ML frameworks, in contrast, are prediction-oriented: they employ automated variable selection, cross-validation for hyperparameter tuning, and non-linear interaction modelling to maximize out-of-sample predictive accuracy [24,26]. Tree-based ensemble methods, such as random forest and XGBoost (Extreme Gradient Boosting), and sequence-modeling architectures, such as long short-term memory (LSTM) networks, can identify complex variable interactions in high-dimensional clinical data that standard regression cannot capture. In NS specifically, ML algorithms can process continuous streams of NICU physiological data to generate dynamic, real-time risk estimates, a functionality that static logistic regression cannot replicate.

Methodological Pipeline for AI Model Development

The development of clinically deployable AI models for NS prediction follows a structured, multi-stage pipeline in which decisions at each stage exert downstream effects on model validity, generalizability, and clinical utility (Table 1).

**Table 1:** The AI model development pipeline for neonatal sepsis prediction Steps 1 through 8 represent the sequential stages from raw data to clinical deployment. Each stage introduces distinct sources of variability that must be controlled to ensure model validity and equitable generalizability [28]. Abbreviations: EHR, electronic health record; NICU, neonatal intensive care unit; MIMIC-III, Medical Information Mart for Intensive Care (third edition); EOS, early-onset sepsis; LOS, late-onset sepsis; XGBoost, Extreme Gradient Boosting; CatBoost, Categorical Boosting; LSTM, long short-term memory; SMOTE, Synthetic Minority Oversampling Technique; AUROC, area under the receiver operating characteristic curve; SHAP, Shapley Additive Explanations; LIME, Local Interpretable Model-agnostic Explanations; EHR, electronic health record

Step	Stage	Description
1	Data Sourcing	EHR extraction (structured and unstructured), NICU bedside monitors, MIMIC-III and institutional repositories
2	Preprocessing	Cleaning, normalization, temporal alignment, handling of missing values, noise reduction
3	Feature Engineering	Perinatal variables (gestational age, birth weight, maternal fever), neonatal biomarkers, dynamic vital-sign trends
4	Algorithm Selection	Random Forest, XGBoost (Extreme Gradient Boosting), CatBoost (Categorical Boosting) for static EOS; LSTM and deep learning for continuous LOS; ensemble approaches for robustness
5	Model Training	Train-test split; k-fold cross-validation; hyperparameter tuning; handling class imbalance (SMOTE, cost-sensitive learning)
6	Validation	Internal validation (AUROC, F1, sensitivity, specificity); external multicentre validation essential for clinical deployment
7	Explainability	SHAP (Shapley Additive Explanations) values, LIME (Local Interpretable Model-agnostic Explanations), attention maps; model cards; clinician-interpretable output displays
8	Clinical Integration	EHR-embedded alerts, tiered thresholds, human-AI collaboration, prospective outcome trials, regulatory approval

Data sourcing and preprocessing: The primary data source for most NICU-based AI models is the electronic health record (EHR), which provides longitudinal records of demographics, laboratory results, vital signs, medications, and clinical documentation. Widely used public repositories, particularly MIMIC-III (released 2016), have enabled model development and cross-institutional benchmarking [27]. However, a substantive practical limitation in this domain, particularly relevant to LMICs, concerns nursing documentation quality: high nursing-to-patient ratios frequently result in incomplete or absent nursing notes, and where documentation is present, accuracy may be compromised by practices such as copied-forward or auto-populated entries. These documentation limitations reduce the clinical signal available to NLP-based models and restrict the generalizability of such approaches to well-resourced settings [28]. Preprocessing steps include data cleaning, normalization, temporal alignment of physiological variables, and management of missing data.

Feature engineering and selection: Feature engineering transforms raw clinical variables into informative inputs. Commonly used features span several domains: perinatal variables, including gestational age, birth weight, mode of delivery, and duration of membrane rupture; neonatal clinical and biochemical parameters, such as Apgar scores (a standardized neonatal assessment tool rated 0-10 at 1 and 5 minutes of life [27,28]), CRP, and complete blood count indices; and dynamic physiological signals including trends in heart rate, respiratory rate, and oxygen saturation. Feature selection methods, including tree-based importance ranking and regularization techniques such as LASSO (Least Absolute Shrinkage and Selection Operator), balance model complexity with interpretability and reduce the risk of overfitting [28].

Algorithm selection: Algorithm choice is guided by data characteristics and the intended clinical application. Gradient boosting frameworks, including XGBoost and CatBoost (Categorical Boosting), handle heterogeneous tabular data efficiently and provide interpretable feature importance outputs. Neural network architectures, particularly LSTM and transformer-based models, are well-suited to capturing temporal dependencies in continuously sampled physiological data. Ensemble approaches combining multiple base learners have demonstrated improved robustness and predictive stability relative to single-model solutions [28].

Model training, validation, and evaluation: Rigorous validation is the fulcrum of clinical credibility. Most published studies employ internal validation via train-test splits or k-fold cross-validation, with performance quantified using AUROC, sensitivity, specificity, positive predictive value (PPV), negative predictive value (NPV), and the F1 score. The F1 score is the harmonic mean of precision and recall (F1 = 2 × (precision × recall) / (precision + recall)) and is particularly informative in imbalanced datasets, such as NS cohorts where sepsis cases constitute a minority class, as it penalizes models that achieve high accuracy through predominantly negative predictions. Precision-recall curves plot the trade-off between PPV (precision) and sensitivity (recall) across all classification thresholds, providing a more discriminating evaluation of model performance than the ROC curve when class imbalance is substantial [28,29]. External validation on independent datasets from geographically and demographically distinct populations remains far less common, yet it is indispensable for demonstrating that a model's predictive capability extends beyond its development cohort. The absence of external validation is the single most cited methodological limitation in current AI-based NS research [29]. It should be noted that the majority of original studies cited in this review did not report 95% confidence intervals for AUROC and other performance metrics. This constitutes a recognized limitation of the current literature, as the absence of confidence intervals precludes full assessment of statistical precision. Where CIs were not reported by source authors, they are not fabricated here, and readers are advised to interpret point estimates accordingly.

Current AI Models: Approaches and Evidence

Static models for early-onset sepsis: EOS prediction models typically leverage maternal and perinatal risk factors available at or shortly after delivery, including gestational age, birth weight, maternal fever, duration of rupture of membranes, and early neonatal laboratory indices. Tree-based ensemble algorithms are the most frequently deployed framework for this indication, given their ability to handle mixed data types and produce clinically interpretable feature importance rankings.

A retrospective Chinese cohort study evaluated six ML algorithms for EOS prediction in NICU neonates, employing SHAP (Shapley Additive Explanations) values to rank predictor contributions [30]. All six models achieved AUROC values exceeding 0.90 on the test set, with CatBoost demonstrating the highest discriminative performance (AUROC 0.975; AUPRC 0.947). Confidence intervals for AUROC were not reported by the source authors; this limits precise estimation of statistical uncertainty around these point estimates. Calibration, decision curve analysis, and learning curve assessment were favourable across the board, lending incremental confidence to the clinical relevance of the CatBoost model [30]. The comparative performance data are summarized in Table 2.

**Table 2:** Comparative performance of six ML algorithms for early-onset neonatal sepsis prediction Abbreviations: AUROC, area under the receiver operating characteristic curve; CatBoost, Categorical Boosting; RF, Random Forest; XGBoost, Extreme Gradient Boosting; MLP, Multilayer Perceptron; SVM, Support Vector Machine; LR, Logistic Regression. The F1 score represents the harmonic mean of precision and recall; values ≥0.80 indicate strong balanced performance in imbalanced datasets. Bold row denotes top-performing model. Source: Tan X et al. [30].

Model	AUROC	F1 Score	Accuracy	Recall	Specificity	Precision
CatBoost	0.975	0.842	0.917	0.889	0.926	0.800
Random Forest	0.968	0.821	0.907	0.852	0.926	0.793
XGBoost	0.952	0.821	0.907	0.852	0.926	0.793
MLP	0.945	0.772	0.880	0.815	0.901	0.733
SVM	0.930	0.755	0.880	0.741	0.926	0.769
Logistic Regression	0.926	0.737	0.861	0.778	0.889	0.700

A complementary study by Youmei and colleagues (2025) developed a 10-algorithm panel using maternal prenatal predictors [31]. XGBoost and Random Forest achieved the highest discrimination (AUROC 0.87; 95% CI not reported by source authors). Using a clinically optimized classification threshold of 0.04, sensitivity reached 92.8% with a specificity of 73.1%, illustrating the trade-off between sensitivity and specificity that must be deliberately calibrated for the intended clinical use case [31].

Kainth and colleagues (2025) evaluated a logistic regression and tree-based ML model using perinatal and early clinical variables, reporting sensitivity of 90% but specificity of only 37-40%, reflecting the inherent challenge of ruling out infection without sacrificing sensitivity in high-risk neonates [32]. The reported external validation dataset in this study was limited in geographic scope and lacked representation from LMIC settings, reducing confidence in cross-contextual generalizability [32]. Collectively, these studies confirm ML feasibility for EOS prediction but are predominantly retrospective, single-centre, and lack external validation, limiting confidence in clinical deployment.

Continuous Physiological Models for Late-Onset Sepsis

LOS prediction models exploit the temporal richness of continuous NICU monitoring data. Heart rate, respiratory rate, oxygen saturation, and motion signals are sampled at high frequency, and ML algorithms detect subtle deviation patterns hours before clinical deterioration becomes apparent.

A retrospective Dutch NICU study demonstrated that a continuous AI model incorporating heart rate, respiratory rate, and oxygen saturation achieved a mean AUROC of 0.875 six hours before critical deterioration, successfully identifying 96.1% of LOS cases (95% CI not reported in source) [33]. Peng and colleagues (2023) further demonstrated that combining heart rate variability (HRV), respiratory, and motion signal features in a multichannel XGBoost classifier achieved an AUROC of 0.88, positive predictive value of 0.80, and negative predictive value of 0.83 during the six hours preceding LOS onset [34]. A multicenter prospective deep learning study by Kallonen and colleagues (2024) using non-invasive biosignals reported an AUROC of 0.810 on the external validation dataset (95% CI not reported), with sensitivity of 83% and specificity of 73%, representing one of the few prospective validation studies available in this domain [35].

Heart Rate Characteristics Monitoring: The HeRO Monitor

The HeRO (Heart Rate Observation) monitor occupies a historically pivotal position in AI-augmented neonatal care. Developed from foundational work on the predictive value of heart rate characteristics (HRC) by Griffin and Moorman (2001) [36], the HeRO monitor calculates an hourly HRC index reflecting the fold increase in sepsis risk over the subsequent 24 hours. This index is derived from two physiological signatures that precede overt infection: (i) reduced heart rate variability (HRV), characterized by a narrowing of normal beat-to-beat fluctuation reflecting autonomic suppression during subclinical infection; and (ii) transient decelerations, brief episodes of slowing in heart rate that interrupt the baseline, analogous to the periodic decelerations observed in foetal distress. Together, these features constitute a characteristic pre-sepsis HRC pattern that is quantified by the HeRO algorithm and displayed as a real-time risk score at the bedside [36,37].

A landmark prospective randomized controlled trial (RCT) enrolling 3,003 very low birth weight (VLBW; birth weight <1,500 g) infants across nine NICUs demonstrated a statistically significant 22% relative reduction in mortality when the HeRO score was displayed to clinical staff (8.1% versus 10.2% in the non-display arm; P = 0.04) [37]. This trial remains the most robust clinical evidence of efficacy for any AI-based NS monitoring tool and establishes the biological plausibility that physiological signal-based AI alerts can translate into a mortality benefit when integrated into existing clinical workflows.

Table 3 summarizes key AI studies on NS prediction.

**Table 3:** Summary of key AI studies on neonatal sepsis prediction Abbreviations: EOS, early-onset sepsis; LOS, late-onset sepsis; AUROC, area under the receiver operating characteristic curve; HR, heart rate; RR, respiratory rate; SpO2, peripheral oxygen saturation; HRV, heart rate variability; PPV, positive predictive value; NPV, negative predictive value; LSTM, long short-term memory; VLBW, very low birth weight (<1,500 g); WBC, white blood cell count; PCT, procalcitonin; CRP, C-reactive protein; RF, Random Forest; XGBoost, Extreme Gradient Boosting; MLP, Multilayer Perceptron; SVM, Support Vector Machine; LR, Logistic Regression; BP, blood pressure; RCT, randomized controlled trial; LMIC, low- and middle-income country

Study	Validation	Algorithm Type	Type	Performance	Key Limitations
Tan et al. 2024 [30]	Retrospective, single-centre	CatBoost, RF, XGBoost, MLP, SVM, LR	EOS	AUROC 0.975; accuracy 0.917; recall 0.889 (no 95% CI reported)	Single-centre; no external validation
Youmei et al. 2025 [31]	Retrospective	XGBoost, RF	EOS	AUROC 0.87; sensitivity 92.8%; specificity 73.1% (no 95% CI reported)	No external validation
Kainth et al. 2025 [32]	Internal + external	LR, tree-based ML	EOS	Sensitivity 90%; specificity 37–40% (no 95% CI reported)	Low specificity; limited LMIC data
Yang et al. 2024 [33]	Retrospective multicentre	Continuous AI model	LOS	AUROC 0.875 at 6h pre-deterioration; 96.1% sensitivity (no 95% CI reported)	Retrospective; infrastructure dependent
Peng et al. 2023 [34]	Retrospective	XGBoost (multichannel)	LOS	AUROC 0.88; PPV 0.80; NPV 0.83 (no 95% CI reported)	Infrastructure dependent; retrospective
Kallonen et al. 2024 [35]	Prospective multicentre	Deep learning (biosignals)	LOS	AUROC 0.810 (external); sensitivity 83%; specificity 73% (no 95% CI reported)	Early-stage; limited sample size
HeRO RCT (Moorman et al. 2011) [37]	Prospective RCT (n=3,003 VLBW)	HRC monitoring	LOS	22% relative mortality reduction; 8.1% vs 10.2% (P=0.04)	Restricted to VLBW; no direct pathogen ID

Comparative Operational Profile: Blood Culture and AI-Based Risk Prediction

Blood culture and AI-based prediction models address fundamentally different clinical questions and should be understood as complementary rather than competing tools. Blood culture is a diagnostic tool that confirms bacteraemia and identifies the causative organism with susceptibility data. No AI model currently replicates this function. AI-based prediction models are risk stratification tools that provide early warning of physiological deterioration preceding clinical diagnosis. The operational comparison in Table 4 should be read in this context.

**Table 4:** Diagnostic comparison: blood culture (reference standard) vs AI-based predictive models This table summarizes the principal operational differences between blood culture and AI-based prediction models in the context of neonatal sepsis. Blood culture data from references [12-14]; AI model performance from references [31,33-35,37]. Abbreviations: AUROC, area under the receiver operating characteristic curve; VLBW, very low birth weight; EOS, early-onset sepsis; LOS, late-onset sepsis; LMIC, low- and middle-income country; EHR, electronic health record; RCT, randomized controlled trial

Parameter	Blood Culture (Reference Standard)	AI-Based Prediction Models
Result turnaround time	24–72 hours	Real-time continuous; early warning 6–12 h pre-onset
Sensitivity	25–40% [12]	Sensitivity up to 92.8% (EOS models); 83% (LOS deep learning) [31,35]
Specificity	Variable; false-positive rate 2.6–18% [13,14]	Specificity 37–73% depending on threshold calibration [31,35]
Predictive window	None (retrospective confirmation only)	6–12 hours before clinical deterioration [33-38]
Infrastructure requirement	Blood sample + laboratory	Continuous NICU monitoring hardware; EHR integration
Pathogen identification	Yes (organism + sensitivity profile)	No; risk prediction only
Clinical evidence level	Gold standard (Lancet 2017 [39,40])	RCT mortality benefit demonstrated for HeRO monitor [37]
LMIC applicability	Limited by sample volume, contamination	Limited by infrastructure and training data availability [40]

Clinical Integration: Translating AI Into NICU Workflows

The transition from algorithmic performance to clinical benefit is neither linear nor automatic. Embedding AI tools into NICU workflows requires attention to system design, human-AI interaction, and the specific clinical contexts in which alerts will be generated and acted upon.

The most appropriate model for AI deployment in the NICU is one of human-AI collaboration: AI functions as a continuous early-warning system that generates evolving risk estimates from integrated EHR, laboratory, and physiological streaming data, while clinicians retain interpretive authority and treatment responsibility [28]. Evidence from the HeRO monitor demonstrates that outcome improvements are greatest when AI-generated signals are presented to clinicians as prompts for reassessment, not as autonomous treatment triggers [36,37]. This principle, algorithmic augmentation of clinical vigilance without displacement of clinical judgment, is foundational to safe deployment.

When appropriately integrated, AI tools offer several tangible benefits: earlier identification of high-risk neonates enabling more timely investigation and antibiotic initiation; and where models with acceptable specificity are deployed under structured clinical governance, support for antibiotic stewardship by identifying neonates at genuinely low risk, enabling earlier antimicrobial de-escalation [15-17]. It bears emphasis, however, that stewardship benefit depends critically on specificity. Models with low specificity (e.g., 37%) used as triggers for antibiotic initiation would increase, not reduce, empirical antibiotic use. The stewardship value of AI lies in its use as a negative predictor ruling out sepsis in monitored neonates with stable physiological trajectories, not as a positive trigger.

Alert fatigue represents a significant operational risk. Poorly calibrated alert systems erode clinician trust, disrupt workflows, and may paradoxically reduce patient safety if alerts are systematically ignored. Mitigation requires longitudinal trend analysis, tiered alert thresholds calibrated to clinical context, and iterative user-centred design informed by clinician feedback [38,39]. A practical consideration for implementation, particularly in resource-constrained settings, is the infrastructure required to support continuous physiological monitoring and EHR integration. The cost of monitoring hardware and EHR systems represents a substantive barrier in LMICs where the NS burden is highest, and must be addressed through investment in health informatics infrastructure and frugal innovation in AI model design.

Barriers to Implementation: Data, Heterogeneity, and Bias

Data quality and availability: The performance ceiling of any AI model is fundamentally constrained by the quality and completeness of its training data. Missing values, high noise levels in EHR-extracted data, and inconsistent documentation practices reduce the signal available to learning algorithms. In LMICs, where the burden of NS is highest, significant proportions of clinical facilities operate with paper-based records or rudimentary digital systems, rendering large-scale structured data collection for model training operationally challenging [40]. This technological asymmetry creates a paradox in which AI development is most advanced in settings where NS is least prevalent, and most limited where the clinical need is greatest.

Definitional heterogeneity and outcome labeling: The absence of a universally accepted, operationally precise definition for NS is a pervasive source of methodological inconsistency. Different centers apply variable clinical, hematological, and microbiological criteria to classify sepsis versus suspected sepsis versus culture-negative sepsis. This heterogeneity produces inconsistent outcome labels across training datasets, undermining comparability between models and reducing the reliability of performance benchmarking.

Pathogen Spectrum and Geographic Bias

AI models trained on data from high-income-country NICUs inherently reflect the pathogen spectrum, antimicrobial resistance patterns, and patient demographics of those settings. In high-income countries, *Group B Streptococcus *(GBS),* Streptococcus agalactiae*), and coagulase-negative Staphylococcus (CoNS) dominate EOS and LOS microbiology, respectively. In contrast, Gram-negative organisms, including *Klebsiella pneumoniae* and *Escherichia coli,* predominate in many LMIC NICUs, where extended-spectrum beta-lactamase (ESBL)-producing strains are disproportionately prevalent [40]. A model trained on North American or European data will therefore encode biological and clinical assumptions that do not transfer to settings with fundamentally different pathogen ecologies, potentially reducing sensitivity precisely where mortality risk is highest. This necessitates context-specific model development, local pathogen surveillance integration, and deliberate inclusion of LMIC cohorts in training and validation datasets.

Translational Gaps

The interpretability problem and explainable AI: Complex ML architectures, particularly deep neural networks, function as computational black boxes: they generate predictions without exposing the reasoning that underpins them. Clinician acceptance of AI-generated recommendations is critically dependent on understanding why a particular alert has been generated [38]. Unexplained predictions may be dismissed, generate inappropriate reliance, or, in cases of error, produce medico-legal ambiguity.

Explainable AI (XAI) methods have been developed to address this limitation by providing post-hoc, human-interpretable explanations for individual model predictions. SHAP values are derived from cooperative game theory and quantify the marginal contribution of each input feature to a specific prediction by calculating the average effect of that feature across all possible feature combinations; in practice, a positive SHAP value for a given variable (e.g., elevated CRP) indicates that this feature pushed the prediction toward the sepsis-positive class, while a negative SHAP value indicates it pushed the prediction away from sepsis [30]. LIME (Local Interpretable Model-agnostic Explanations) provides an alternative approach by approximating the behaviour of the complex model locally around a specific prediction using a simpler, interpretable linear model, thereby identifying which features most influenced that particular case's output [38]. Both methods constitute essential components of any AI system intended for clinical deployment, providing clinicians with feature-level justification for alert generation. Regulatory frameworks are increasingly mandating explainability as a condition for approval.

Workflow integration: Technical performance in a research environment does not guarantee successful adoption in clinical practice. EHR integration requires interoperability standards (such as HL7 FHIR; Health Level Seven Fast Healthcare Interoperability Resources), low-latency data pipelines, and system architecture resilience that many NICU IT infrastructures currently lack. Clinical usability, including the design of alert interfaces, user experience, and clinician training requirements, further determines whether AI tools are adopted or abandoned after deployment. Human factors research specifically targeting AI alert integration in NICU settings is markedly underrepresented in the literature.

The prospective evidence gap: The most fundamental translational limitation is the scarcity of prospective evidence demonstrating that AI-based NS prediction actually improves hard clinical outcomes. The overwhelming majority of published AI studies are retrospective, evaluating algorithmic performance on historical datasets without assessing clinical impact [29]. In the absence of prospective, preferably randomized, evidence demonstrating reductions in sepsis-related mortality, antibiotic exposure, or NICU length of stay, regulatory approval and clinical adoption are difficult to justify. The HeRO monitor RCT stands as a rare counterexample and should serve as a methodological template for future prospective AI trials [37].

Ethical, Legal, and Regulatory Considerations

Algorithmic bias and equity: AI models learn from historical data that may encode systemic biases related to race, socioeconomic status, or geography. If training populations over-represent certain demographic groups, the resulting model may systematically underperform for others. In neonatal care, this risk carries direct mortality implications for the most vulnerable infants in the most underserved communities. Bias auditing, the systematic evaluation of model performance across demographic strata, and the deliberate inclusion of diverse training populations are necessary safeguards against the amplification of existing health inequities [40,41].

Accountability and liability: When a clinical decision informed by AI leads to patient harm, determining accountability is legally and ethically complex. Current regulatory frameworks in most jurisdictions do not clearly delineate responsibility between the AI developer, the institution deploying the tool, and the clinician who acted on its output. Establishing clear governance structures that assign accountability, mandate AI transparency, and require documentation of AI-informed decisions in the medical record is a prerequisite for responsible clinical deployment [42].

Data privacy and consent: Neonatal health data are among the most sensitive categories of personal information, encompassing genetic risk, maternal health history, and socioeconomic indicators. Data governance frameworks must comply with applicable privacy legislation, including the General Data Protection Regulation (GDPR) in the European Union and the Health Insurance Portability and Accountability Act (HIPAA) in the United States, and must address the specific vulnerabilities of a patient population that cannot provide consent. Parental consent processes, data minimization principles, and secure federated learning architectures that enable model training without centralizing raw patient data are important tools in this context.

Regulatory pathways: AI-based clinical decision-support tools are increasingly regulated as Software as a Medical Device (SaMD) by bodies including the US Food and Drug Administration (FDA) and the European Medicines Agency (EMA). Pre-market evaluation requires demonstration of analytical validity, clinical validity, and clinical utility. Regulatory pathways for adaptive AI clinical decision-support tools remain heterogeneous, evolving, and incompletely standardized at the global level. In the United States, the FDA's predetermined change control plan (PCCP) framework provides a mechanism for approving continuously learning AI models while maintaining regulatory oversight; the EMA and the UK Medicines and Healthcare products Regulatory Agency (MHRA) have each issued guidance on AI-based medical devices, though frameworks continue to be refined and harmonized internationally. Developers are advised to engage with applicable regulatory bodies early in the development process, as requirements differ materially by jurisdiction and continue to evolve. Translation from research tool to approvable clinical product requires early and iterative engagement with these pathways [42].

Global Health Perspective and Equity Implications

The global distribution of NS burden is inverted relative to the global distribution of AI research capacity. LMICs, where 95% of neonatal deaths occur, account for a small fraction of published AI models [43]. Without deliberate corrective action, the diffusion of AI-driven diagnostics may amplify rather than attenuate global health inequity, conferring technological benefit on populations with the lowest absolute risk while leaving the highest-burden settings further behind.

Addressing this disparity requires a multi-pronged strategy. Federated learning architectures enable collaborative model training across distributed institutional datasets without transferring sensitive patient data, facilitating participation by LMIC institutions with limited data infrastructure. Transfer learning approaches, in which models pre-trained on large high-income-country datasets are fine-tuned on smaller LMIC cohorts, can partially bridge the data volume asymmetry [40]. Open-source model repositories, standardized minimum clinical datasets for NS research, and North-South research partnerships involving co-investigators from high-burden countries are further mechanisms for building equitable AI capacity.

Resource-constrained settings also demand context-appropriate model design. Models dependent on continuous high-frequency physiological monitoring, or on laboratory biomarkers not routinely available in district hospitals, are functionally inapplicable regardless of algorithmic performance. AI for global NS must prioritize frugal innovation: models trained on clinically accessible, low-cost inputs that are applicable in resource-limited settings while maintaining meaningful predictive performance.

Emerging Frontiers and Future Research Directions

Multi-omics integration: The integration of genomic, transcriptomic, proteomic, and metabolomic data with clinical variables represents a frontier for next-generation NS prediction models. Host genomic susceptibility loci, inflammatory gene expression signatures, and microbiome compositional patterns have demonstrated associations with sepsis risk and severity [44].

Large language models (LLMs) and unstructured data: LLMs offer a pathway to extract clinically relevant information from unstructured EHR text, including nursing notes, clinical summaries, and procedural documentation. Their application to NS prediction remains in early development. A substantive limitation in LMICs is that nursing notes are frequently absent, given high nursing-to-patient ratios and limited documentation infrastructure. Where nursing notes are present, accuracy may be further compromised by practices such as copied-forward or auto-populated entries, a documentation limitation that significantly reduces the signal available to LLM-based extraction approaches and restricts their applicability to well-resourced settings. This limits the generalizability of LLM-based approaches to settings with adequate and accurate nursing documentation.

Reinforcement learning for treatment Optimization: Beyond prediction, reinforcement learning algorithms can learn optimal treatment policies by simulating clinical decision environments. In the context of NS, RL could inform antibiotic selection, dosing duration, and de-escalation timing. The time-critical nature of NS and the ethical constraints of off-policy learning in critically ill neonates must be resolved before clinical deployment.

Continuous learning and adaptive models: Static models trained on historical data will inevitably experience performance degradation as pathogen spectra and clinical practices evolve. Continuously learning AI systems capable of updating their parameters in response to incoming data require urgent development of regulatory frameworks for adaptive clinical AI [42,44].

Priority research agenda: The field would benefit from: prospective, multicenter, randomized trials powered to detect differences in clinical outcomes; development of validated, globally applicable minimum datasets for NS AI research; creation of LMIC-representative open training repositories; and head-to-head comparisons of AI-assisted versus standard care decision-making in real-time NICU environments.

Table 5 lists the barriers to AI implementation in NS prediction.

**Table 5:** Barriers to AI implementation in neonatal sepsis prediction and proposed mitigations Abbreviations: EHR, electronic health record; LMIC, low- and middle-income country; SHAP, Shapley Additive Explanations; LIME, Local Interpretable Model-agnostic Explanations; RCT, randomized controlled trial; SaMD, Software as a Medical Device; FHIR, Fast Healthcare Interoperability Resources; XAI, explainable artificial intelligence; GBS, Group B Streptococcus. References for individual rows: data quality [40]; heterogeneity [29]; pathogen bias [40]; interpretability [30,38]; alert fatigue [38,39]; evidence gap [37]; algorithmic fairness [40,41]; regulatory gaps [42].

Domain	Key Challenge	Proposed Mitigation
Data Quality	Missing values, noise in EHRs, limited digitization in LMICs, paper-based records	Federated learning frameworks; standardized minimum dataset requirements; investment in LMIC health informatics infrastructure
Heterogeneity	No universal definition of neonatal sepsis; variable outcome labelling across centres	Adoption of consensus definitions (e.g., Sepsis-3 neonatal adaptations); multi-institutional data harmonization initiatives
Pathogen Spectrum Bias	Models trained in high-income settings may not generalize to LMICs with distinct pathogen profiles (Gram-negative predominance vs Gram-positive)	Context-specific model development; international open-source datasets; transfer learning from large to small datasets
Black-Box Interpretability	Clinician distrust of opaque deep learning outputs; medico-legal concerns	Explainable AI (XAI): SHAP values, LIME; model cards detailing performance across subgroups
Alert Fatigue	Excessive or poorly calibrated alarms erode clinical trust and disrupt workflow	Tiered alert thresholds; longitudinal trend analysis; user-centered design of alert interfaces [38,39]
Prospective Evidence Gap	Majority of studies are retrospective; real-world clinical impact unproven	Pragmatic prospective RCTs with hard clinical endpoints (mortality, length of stay, antibiotic days)
Algorithmic Bias and Fairness	Training data may encode demographic or socioeconomic biases	Bias auditing; diverse and representative training cohorts; fairness-aware algorithms [40,41]
Regulatory and Liability Gaps	Regulatory pathways for adaptive AI clinical decision support tools remain heterogeneous, evolving, and incompletely standardized globally	Engagement with the FDA Software as Medical Device (SaMD) framework, EMA, and MHRA guidance; pre-market and post-market surveillance protocols [42]

Study Limitations

The majority of existing AI models for NS prediction are developed using retrospective, single-center datasets, which limit their external validity and may not accurately reflect diverse patient populations or healthcare settings, particularly in LMICs. Additionally, the present review is itself subject to the limitations inherent in a structured narrative synthesis: only three databases were searched (PubMed, Google Scholar, IEEE Xplore); Embase, Cochrane CENTRAL, and Scopus were excluded, which may have resulted in the omission of relevant studies. The final study selection was undertaken narratively without dual independent screening or a formal risk-of-bias assessment, introducing potential for selection and extraction bias. Furthermore, the majority of original studies cited in this review did not report 95% confidence intervals for AUROC and related performance metrics, precluding robust assessment of statistical precision across the evidence base; this limitation is acknowledged throughout the results sections where applicable. There is also a significant lack of prospective, multicenter clinical trials evaluating the real-world effectiveness and impact of AI-based sepsis prediction tools on patient outcomes, hindering the translation of these models into routine clinical practice.

## Conclusions

Artificial intelligence offers a genuinely transformative opportunity to supplement the diagnostic tools that have long challenged early, accurate detection of neonatal sepsis. The evidence reviewed here must, however, be read with appropriate nuance. Blood culture, biochemical markers, and clinical assessment remain essential and effective tools when deployed by experienced clinicians with appropriate contextual knowledge; their limitations are operational rather than categorical, and AI is best positioned as an additional monitoring layer that compresses the window of clinical uncertainty rather than as a replacement for existing practice. Machine learning models for EOS prediction, particularly ensemble tree-based classifiers, including CatBoost and XGBoost, and continuous physiological monitoring algorithms for LOS consistently demonstrate strong discriminative performance in retrospective settings. The HeRO monitor RCT provides proof of principle that physiological AI signals, integrated thoughtfully into clinical workflows, can produce measurable mortality benefits. Nevertheless, the path from algorithmic performance to population-level clinical benefit remains incompletely mapped.

Key constraints include a concentration of retrospective, single-center studies with limited external validity; a critical shortage of prospective evidence linking AI predictions to hard clinical outcomes; the absence of equitable representation of LMIC contexts where Gram-negative pathogen predominance fundamentally distinguishes the microbial landscape from settings in which most models were trained; unresolved questions of specificity and antibiotic stewardship; the near-universal absence of 95% confidence intervals in reported performance metrics, which limits precise statistical interpretation of published findings; and gaps in interpretability, accountability, and regulatory governance. Overcoming these barriers demands methodological advances including the deployment of SHAP and LIME within explainable AI frameworks, and deliberate interdisciplinary and international collaboration among neonatologists, data scientists, biomedical engineers, clinical ethicists, and global health researchers. The ultimate goal is an equitable, globally applicable partnership in which AI manages high-volume pattern recognition and continuous physiological vigilance, and clinicians apply contextual judgment, ethical reasoning, and therapeutic authority to the most vulnerable patients in the most underserved settings.
